# Editorial: Interactions Between Bioactive Food Ingredients and Intestinal Microbiota

**DOI:** 10.3389/fmicb.2022.902962

**Published:** 2022-04-22

**Authors:** Zheng Ruan, Fengjie Sun, Xiaodong Xia, Guodong Zhang

**Affiliations:** ^1^School of Food Science and Technology, Nanchang University, Nanchang, China; ^2^School of Science and Technology, Georgia Gwinnett College, Lawrenceville, GA, United States; ^3^School of Food Science and Technology, Dalian Polytechnic University, Dalian, China; ^4^Department of Food Science, University of Massachusetts Amherst, Amherst, MA, United States

**Keywords:** Bioactive Food Ingredients, gut health, gut microbiota, polyphenol, microbe-microbe interactions, metabolomics

With the rapid advancement in the various “omics” technologies, a wide range of strategies (e.g., the application of dietary nutrients) have been extensively explored worldwide to improve the human health (Hasin et al., 2017; Nayak et al., 2021; Si et al., 2021). Numerous studies have shown that the microorganisms that colonize the gastrointestinal tract play important roles in not only the digestion and absorption of dietary nutrients, but also the regulation of various biological activities in hosts, e.g., metabolism, immunity, and intestinal barrier function (Wang et al., 2019: Han et al., 2021; Singh et al., 2021). Furthermore, multiple lines of evidence have suggested that food containing nutrients and polyphenols plays critical roles in determining the composition and function of the gut microbiota, ultimately directly or indirectly mediating the host's physiological activities (Ray and Mukherjee, 2021). To date, the molecular mechanisms regulating the interactions among the food nutrients/prebiotics, gut microbiota, and host health remain largely unclear.

It is commonly believed that the dietary approaches are effective to improve human health by the precision microbiome modulation (Deehan et al., 2020). Therefore, clear understanding of the molecular mechanisms that regulate the gut microbiota and its metabolic activities by dietary nutrients is beneficial in developing the dietary strategies. Delighted by these exciting achievements in the studies of the interactions among food nutrients, gut microbiota, and human health, we strongly believe it is necessary and important to further strengthen the current contributions from scholars worldwide particularly in the areas of the interactions between food nutrient and gut microbiota and the roles played by these interactions in the development of dysbiosis and low-grade inflammation during the intestinal barrier dysfunction and metabolic disorders in hosts. We here summarize the main results of the 21 out of a total of 27 submissions contributed by over 170 contributors accepted for publication in our Research Topic entitled “Interactions between Bioactive Food Ingredients and Intestinal Microbiota” in the journal *Frontiers in Microbiology*.

Briefly, these following topics are widely explored by the studies in this Research Topic: modulation of gut microbiome composition and function by food nutrients in metabolic disorders or inflammation-related intestinal diseases, the possible mechanisms for the microbial metabolites derived from dietary in mediating the physiological activities in hosts, the microbe-microbe interactions with the nutritional intervention, including the microbial communication and signaling, interactions among diet, gut microbiota, and gut health/host metabolism, development of nutritional strategies to precisely modulate gut microbiome and gut health. Most of the contributions collected in this Research Topic are performed based mainly on animal models, i.e., nine articles on pigs, nine on mice or rats, one on chicken, and one on bacteria, using various well-established “omics” analyses, as well as one review article. We here summarize the main results derived from these contributions.

First, in the nine contributions based on pigs, various substances have been supplemented in diet to significantly increase the growth performance and immunity and alter the composition of the gut microbiota. For example, Li et al. have shown strong evidence to suggest that as an indispensable essential micronutrient for humans and other animals, the organic selenium (i.e., 2-hydroxy-4-methylselenobutanoic acid) is capable of significantly increasing the antioxidant capacity and immune function and changing the intestinal microbiota in gilts, while the expression patterns of various genes related to selenoproteins and various cytokines are significantly regulated. This study demonstrates the potential applications of organic selenium in gilt and pig productions. Similarly, Diao et al. have shown the strong effects of dietary zinc on the growth performance and gut health in weaned piglets, as suggested by various growth performance factors and significantly regulated expressions of digestion and absorption related genes. Furthermore, several types of plant products are revealed to show the enhancing effects on the growth performance in piglets. For example, Li et al. have used the mixture of five fermented traditional Chinese medicinal herbs, including *Codonopsis pilosula* (Dangshen), *Radix astragalus* (Huangqi), *R. isatidis* (Banlangen), *R. paeoniae alba* (Baishao), and *Atractylodes macrocephala* (Baizhu), to significantly increase the growth performance and change the intestinal microbiota in piglets, while the mixture is also capable of increasing the total antioxidant capacity and decreasing the damage caused by H_2_O_2_ to the tight junction proteins of the porcine small intestinal epithelial cell line. Similarly, Li et al. have revealed the strong effects of the dietary fermented mao-tai lees (i.e., a by-product of Maotai liquor) on the growth performance, plasma metabolites, including various types of amino acids, and intestinal microbiota and metabolites of weaned piglets. It is interestingly noted that the amino acid metabolism is enhanced by the mao-tai lees without affecting the growth performance in the weaned piglets. Furthermore, Luo et al. and Gu et al. have shown that another two types of fermented plant products, i.e., alfalfa meal and cottonseed meal, have significantly improved the growth performance, intestinal health and immunity in weaned pigs. For example, the expressions of genes involved in immunity and intestinal barrier are significantly increased in piglets fed with alfalfa meal. These studies have provided the solid evidence to support the partial use of fermented plant products as the dietary component to ultimately reduce the consumption of grain. Moreover, Luo et al. have demonstrated that different types of dietary fibers (i.e., peahull fiber, oat bran, and the mixture of both) are revealed to show differential effects on intestinal health of growing pigs, as suggested by the varied taxonomic structures in the gut microbiota and immune-related indices. Additionally, Yu et al. have explored the alternation of the intestinal microbiota in porcine fed with the dietary manno-oligosaccharide, which is a type of prebiotic generally derived from plants or yeasts, providing novel insights into the molecular mechanisms regulating the interactions between the health and the intestinal microbiota modulated by the manno-oligosaccharide. By metagenomic sequencing, Hu et al. have investigated the effect of berberine, which is generally used as an antibacterial medicine, on the intestinal microbiome in weaned piglets. Their results have revealed significant improvements in composition, abundance, structure, and function of gut microbiome in the weaned piglets treated with berberine, providing strong evidence to support the application of berberine in human and animal health.

Second, in another nine articles based on mice or rats, various types of plant products (e.g., tea), extracts from both plants and fungi (i.e., polysaccharides, flavones, polyphenol (i.e., ellagic acid), and gastrodin), and bacteria have been used to enhance the health performance in mouse or rat models. For example, Wang et al. and Feng et al. have investigated the effects of Tibetan tea and green tea, respectively, on the gut microbiota in mouse models. Specifically, their results have revealed the protective effects on the ulcerative colitis in mice, with the expressions of genes involved in the pathways of inflammation and immune system significantly regulated, while both the β-Carotin and green tea powder have shown alleviating effects on the symptoms of gouty arthritis and have improved the gut microbiota in mice, probably attributing to the high contents of dietary fiber in both substances. Yang et al. and Fu et al. have used the plant polysaccharides derived from *Lycium barbarum* and *Portulaca oleracea* to reduce the obesity in mice fed with high-fat diet and to modulate the intestinal microbiota and inhibit the reproduction of pathogenic bacteria in aged rats, respectively. Their results have revealed that these polysaccharides may be the potential sources of prebiotics to improve the lipid metabolism and intestinal diseases. Additionally, the functions of another two types of plant extracts, i.e., gastrodin and ellagic acid, have been investigated by Wang et al. and Xu et al. in mouse models, respectively. As a type of phenolic glycoside, gastrodin is the main bioactive constituent of *Rhizoma gastrodiae*, while ellagic acid is generally found in many types of fruits and vegetables. Specifically, gastrodin has been revealed to show both the analgesic and anxiolytic effects and influence both the ferroptosis and jejunal microbiota in mice, while the ellagic acid is capable of enhancing the growth of mice, promoting the intestinal development, increasing the antioxidant capacity, and regulating the intestinal microbiota in mice. Furthermore, Xu et al. and Xu et al. have investigated the effects of the polysaccharides and flavones derived from the fungal species *Scorias spongiosa* and *Morchella importuna* on the intestinal microbiota in mice, respectively. Their results have revealed that although the polysaccharides derived from *Scorias spongiosa* have shown no significant effects on the growth performance of mice, the polysaccharides benefit the intestinal health in mice through a molecular mechanism with the involvement of elevated antioxidant and anti-inflammatory activities and significantly regulated intestinal microbiota, while the polysaccharides derived from *Morchella importuna* have shown protective effects on the damage in mice induced by the dextran sulfate sodium through the mechanism with the inhibition of the nuclear factor kappa B and the activation of the nuclear factor erythroid 2–related factor 2 as well as the regulation of intestinal microbiota. Moreover, Xie et al. have shown that *Lactiplantibacillus plantarum* AR113 is capable of significantly accelerating the liver regeneration by regulating the gut microbiota and plasma glycerophospholipid in rats.

Third, in the only contribution based on chicken in this Research Topic, Chen et al. have performed the multi-omics analysis in chickens with the vaginal administration of *Bacteroides fragilis*. Their results have provided a theoretical basis for the application of *B. fragilis* as a potential probiotic in livestock and poultry production.

Fourth, Fan et al. have investigated the effects of osmotic pressure on the translation efficiency of *Lactobacillus rhamnosus* based on the ribosome profiling analysis at the genomic level. As a probiotic strain, *L. rhamnosus* has been widely used in various types of fermented and functional food production, playing an important role in modern biotechnological fermentation processes. The results of ribosome profiling analysis have shown that *L. rhamnosus* responds to osmotic stress by translation regulation and controls the balance between survival and growth of cells by transcription and translation.

Lastly, in the only review contribution collected in this Research Topic, Hong et al. have investigated the well-known antioxidant activities of tea polyphenols to further explore the metabolic pathways with the involvement of host-microbiota interactions. They have established the significance of several intestinal metabolites (i.e., 5-hydroxytryptamine and short-chain fatty acids) in the assessment of the tea polyphenol-mediated chronic brain diseases. This review provides novel insights into the molecular mechanisms regulating the interactions between the chronic neurological disorders and the microbial metabolites.

In summary, the collection of these front-line contributions has evidently shown not only the main advancements in these areas covered in this Research Topic to potentially make significant improvement in human health, but also the future directions with the challenges displayed for the scholars to pursue in their investigations. With the discoveries of many types of substances showing the capability of enhancing the growth performance, immunity, and the intestinal microbiota in piglets as well as the rapidly developing “omics” technologies, it is optimistically foreseen that in the near future, we would see another unprecedented wave of major achievements obtained in these areas covered in this Research Topic closely related to human health.

## Author Contributions

ZR and FS wrote the original draft. XX and GZ edited and reviewed the manuscript. All authors contributed to the article and approved the submitted version.

## Conflict of Interest

The authors declare that the research was conducted in the absence of any commercial or financial relationships that could be construed as a potential conflict of interest.

## Publisher's Note

All claims expressed in this article are solely those of the authors and do not necessarily represent those of their affiliated organizations, or those of the publisher, the editors and the reviewers. Any product that may be evaluated in this article, or claim that may be made by its manufacturer, is not guaranteed or endorsed by the publisher.
